# Land-surface initialisation improves seasonal climate prediction skill for maize yield forecast

**DOI:** 10.1038/s41598-018-19586-6

**Published:** 2018-01-22

**Authors:** Andrej Ceglar, Andrea Toreti, Chloe Prodhomme, Matteo Zampieri, Marco Turco, Francisco J. Doblas-Reyes

**Affiliations:** 1 European Commission, Joint Research Centre, via Enrico Fermi 2749, 21027 Ispra, Italy; 2 Barcelona Supercomputing Center (BSC), c Jordi Girona 29, 08034 Barcelona, Spain; 3 University of Barcelona, Av. Diagonal 647, 08028 Barcelona, Spain; 4 Institució Catalana de Recerca i Estudis Avançats (ICREA), Passeig de Lluis Companys 23, 08010 Barcelona, Spain

## Abstract

Seasonal crop yield forecasting represents an important source of information to maintain market stability, minimise socio-economic impacts of crop losses and guarantee humanitarian food assistance, while it fosters the use of climate information favouring adaptation strategies. As climate variability and extremes have significant influence on agricultural production, the early prediction of severe weather events and unfavourable conditions can contribute to the mitigation of adverse effects. Seasonal climate forecasts provide additional value for agricultural applications in several regions of the world. However, they currently play a very limited role in supporting agricultural decisions in Europe, mainly due to the poor skill of relevant surface variables. Here we show how a combined stress index (CSI), considering both drought and heat stress in summer, can predict maize yield in Europe and how land-surface initialised seasonal climate forecasts can be used to predict it. The CSI explains on average nearly 53% of the inter-annual maize yield variability under observed climate conditions and shows how concurrent heat stress and drought events have influenced recent yield anomalies. Seasonal climate forecast initialised with realistic land-surface achieves better (and marginally useful) skill in predicting the CSI than with climatological land-surface initialisation in south-eastern Europe, part of central Europe, France and Italy.

## Introduction

Advancing the understanding on how climate variability and extremes influence crop production provides the basis to develop an integrated seasonal crop yield forecasting system^1–4^. Seasonal climate forecasts represent an important tool to inform end-users with greater accuracy^5–8^ by also providing a quantification of uncertainties, a key aspect in the decision-making process^9,10^. Even though additional value for agricultural applications in several regions of the world has been shown^11^, seasonal climate forecasts have been faced with numerous challenges to adequately respond to the end-users expectations in impact sectors such as agriculture. The low skill in variables such as precipitation in key regions like Europe and the lack of understanding of the inherent dependency between the forecast time for a skilful forecast and the spatial scale^12^ have limited the applicability of these long-term forecasts. The still challenging prediction of extreme events (such as the 2003 heat wave) in the extra-tropical regions^13^ has also contributed.

Nevertheless, new emerging findings show the potential for a better understanding of the spatio-temporal features of extremes, along with their precursors^14–16^. Although the skill of seasonal forecasts is generally limited in Europe, there are regions and seasons where significant skill appears as a result of processes like the ongoing climate change and/or soil processes, among others^17^. Rainy winter/spring seasons in southern Europe have been shown to inhibit hot summer days, whereas dry conditions are followed by either high or low frequency of hot days^14^. Soil moisture plays an important role, and land-surface initialisation can have a substantial impact on sub-seasonal to seasonal forecast quality in Europe^16,18^.

Here we explore the implications of land surface initialisation in seasonal climate prediction for maize crop yield forecasting in Europe. We further develop a combined stress index (*CSI*) based approach^19^ to integrate the impact of drought and heat stress, which are among the most important growth-limiting factors during the flowering and grain filling period^20^, on maize yield inter-annual variability. Therefore, we show how realistic land surface initialisation of seasonal climate prediction^16^ can provide skill for predicting the *CSI* and consequently maize yield in Europe.

## Results and Discussion

Several recent studies have shown that drought events are compounded with prolonged high temperatures^21^, two key stress factors affecting crop yield variability. The *CSI*^19^-based model, applied here to capture the impact of drought and heat stress events, shows good predictive performance (in terms of *Q*^2^, see Methods) in reproducing the maize yield variations under observed climate conditions in most countries of central, western and south-eastern Europe (Table 1). Predictive performance ranges between 22% in Belgium and 79% in Germany, averaging to 53% over all countries where it is statistically significant. However, no significant relationship is identified in Portugal, Spain, Greece and Turkey (Fig. S1). In these countries, the impact of summer drought and heat stress on maize yields is limited due to the predominant irrigation^22,23^ stabilising national yield and reducing the inter-annual variability. In this group of countries, the decadal trend plays a dominant role when it comes to yield prediction^4^. The derived *CSI* model is also not significant in the Netherlands (Fig. S2). Indeed, heat stress does not seem to play an important role in triggering maize yield losses in the Netherlands^24^ moreover, capillary rise from shallow groundwater levels can alleviate drought stress impacts^25^.

**Table 1:** Predictive performance (*Q*^2^) of country specific *CSI* models. Bold denotes statistically significant values.

Country	*Q* ^2^	Country	*Q* ^2^	Country	*Q* ^2^
Portugal	0.05	Poland	**0.52**	Romania	**0.68**
Spain	0.02	The Czech Republic	**0.58**	Bulgaria	**0.67**
France	**0.72**	Slovakia	**0.41**	Macedonia	**0.37**
Belgium	**0.22**	Austria	**0.39**	Greece	0.04
Netherlands	0.04	Slovenia	**0.59**	Italy	**0.47**
Germany	**0.79**	Hungary	**0.42**	Turkey	0.04

In several countries of southern and central Europe, the *CSI* analysis reveals that heat stress has generally more pronounced influence on maize yield inter-annual variability than drought (Fig. S3). In Italy, maize yields exhibit the highest sensitivity to heat stress, while the drought stress sensitivity is substantially lower. Different drivers as well as their interaction need to be considered to understand this complex response, irrigation being among the most important. For instance, 40% of maize cropland in Italy is irrigated^26^, resulting in reduced sensitivity to drought. Irrigation decreases (up to a certain extent) also the impact of heat stress on maize growth by lowering the canopy temperature during daytime^27^. On the other side, higher night temperatures (often associated with heat waves) increase the rate of leaf senescence^28^. Maize yield is more sensitive to heat stress in many countries with low share of irrigated maize cropland, such as Germany, Romania, Hungary and Macedonia. In France, Slovakia, Austria and Bulgaria, the relative importance of drought is comparable to heat stress. As for France, this confirms previous findings^3^ reporting a relative increase of heat stress effects and decrease of rainfall importance due to irrigation and technological improvements in the last two decades. Contrarily, Slovenia and the Czech Republic exhibit higher sensitivity to drought stress.

**Figure 1:**
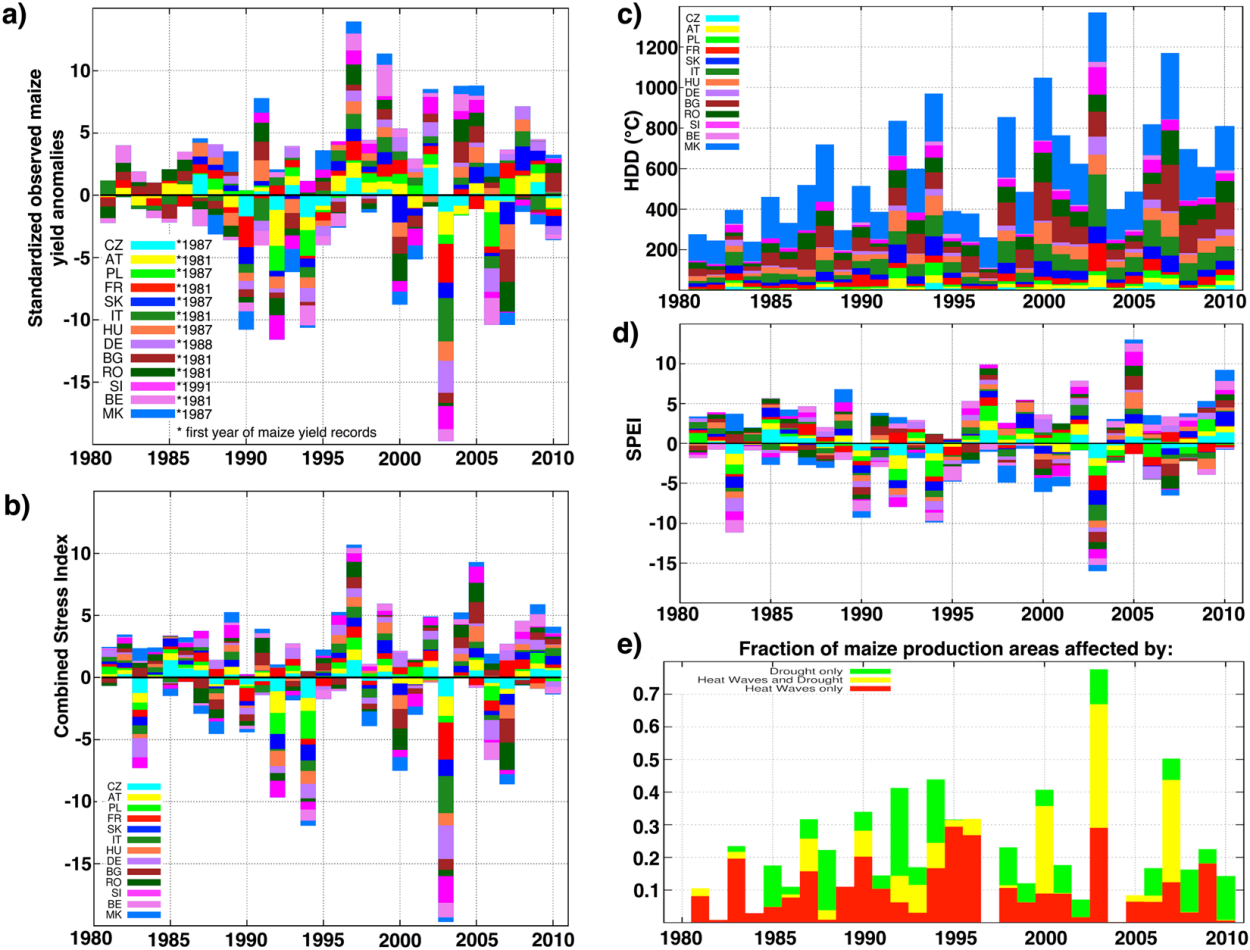
Combined Stress Index and maize yield in Europe from 1981 to 2010. (**a**) Time series of standardised observed maize yield anomalies. In each year, the country based values are superimposed onto each other to form a stacked barplot; colours are associated with countries. Asterisks denote the first year of available maize yield records. The time series on graphs (**b**–**e**) are calculated for all countries during the entire period 1981–2010. (**b**) *CSI* time series are derived from the *E* − *OBS* observational dataset. In each year, the *CSI* values are superimposed onto each other to form a stacked barplot; colours are associated with countries. (**c**) Time series of heat degree days (*HDD*) in country-specific maize crop growing areas. (**d**) Time series of standardised precipitation evapotranspiration index (*SPEI*) in country-specific maize crop growing areas. (**e**) Fraction of maize production areas in countries with significant *CSI* models (see Table 1), affected by drought only ($SPE{I}_{opt,t}^{\ast }$ less than −1 and $HD{D}_{JJA,t}^{std{,}^{\ast }}$ less than 1), heat wave only ($SPE{I}_{opt,t}^{\ast }$ higher than −1 and $HD{D}_{JJA,t}^{std{,}^{\ast }}$ higher than 1) or combined effect of heat and drought ($SPE{I}_{opt,t}^{\ast }$ less than −1 and $HD{D}_{JJA,t}^{std{,}^{\ast }}$ higher than 1).

Since 1990 in maize agricultural land, the *CSI* shows an increase in both the inter-annual variability and the intensity of the events (Fig. 1b). The same behaviour characterises both the heat stress and the drought stress events taken separately (Fig. 1c,d). Exceptionally negative *CSI* values can be observed in several years when countries experienced substantial negative yield anomalies: 1992, 1994, 2000, 2003 and 2007 (Fig. 1a,b). In line with increasing inter-annual variability, the *CSI* also shows higher positive anomalies after 1990, e.g. in 1997 and 2005. These two years are mainly characterised by the absence of heat and drought stress across Europe. There is not a clear tendency in the maize areas affected by drought, heat stress or both together (Fig. 1e). However, concurrent drought and heat stress events seem to be more relevant for the recent negative yield anomalies in 2000, 2003 and 2007.

**Figure 2:**
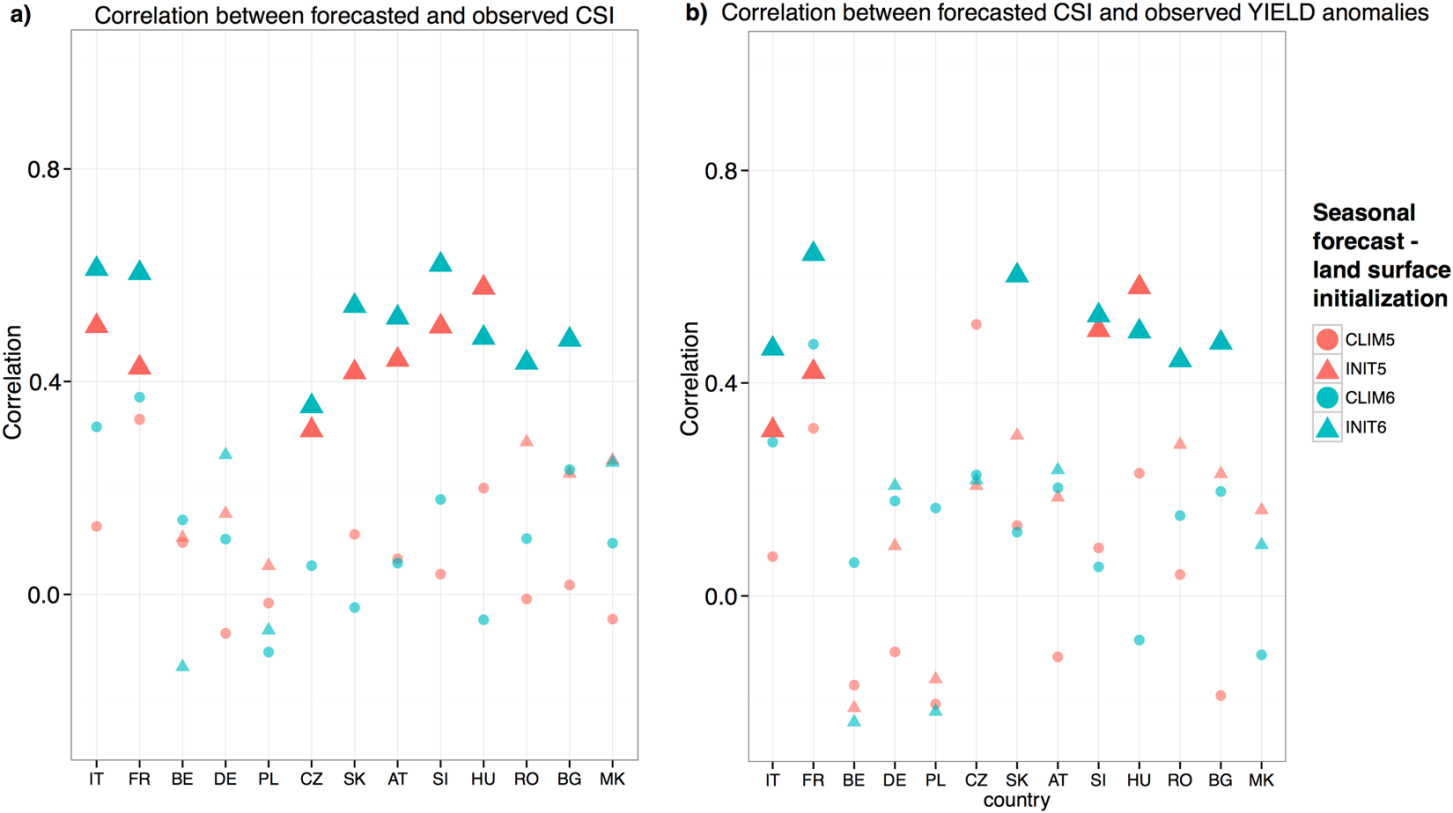
Effects of land-surface initialisation in the seasonal climate forecast. (**a**) Pearson correlation between the *CSI* derived from *E* − *OBS* observational data and different seasonal forecast experiments using climatological (*CLIM*05 and *CLIM*06 for May and June runs, respectively) and realistic land-surface initialisation (*INIT*05 and *INIT*06 for May and June runs, respectively). The forecast *CSI* is calculated from initial condition ensemble average for each experiment. The significance of correlation is indicated by the size of symbols; larger size indicates significant correlations (p < 0.05), whereas smaller size indicates non-significant correlations. (**b**) same as (**a**) but correlation is calculated between the forecasted *CSI* and the observed standardised maize yield anomalies (*Y*^*std*,*^).

Country based *CSI* models, derived from the full observational time series of maize yields, are further used to assess the predictability of yield anomalies with seasonal forecast. Countries not having a significant *CSI* model are excluded from this analysis (Table 1). Initialisation with realistic land-surface for seasonal forecasts performed with EC-Earth2.3^16,29^ in May and June leads to better seasonal prediction of warm extremes and heat waves, and therefore also to *CSI* forecasts better capturing the observed inter-annual variability of both *CSI* and maize yield anomalies (Fig. 2a,b). Climatological land-surface initialisation in the seasonal forecasts (*CLIM*05: May and *CLIM*06: June) generally fails to reproduce the relevant drought and heat stress patterns in summer. The realistic initialisation leads to a substantial performance improvement in France, Italy, central and south-eastern Europe, also in terms of crop yield anomaly prediction. In most of these countries, significant correlation for the *CSI* is found for the forecasts started in both May (*INIT*05) and June (*INIT*06). This is highly relevant as the beginning of May roughly coincides with the emergence or early vegetative stages of grain maize. Contrarily, no significant improvement is observed in Belgium, Germany and Poland (Fig. 2a,b). This seems to point to latitude dependent maize yield forecast skill improvement, most significantly below 50°*N*.

The impact of land-surface initialisation is spatially variable for the heat stress component of the *CSI* and country specific for the drought component. Overall, a more positive impact of soil initialisation can be observed for the heat stress events with respect to drought events (Fig. S4b). This holds also for Germany, where forecasted *CSI* is not significantly correlated with the observed counterpart in any of the forecasts; nevertheless, the forecast of the heat stress events shows skill in both *INIT*05 and *INIT*06 experiments. As for the drought component of the *CSI*, the realistic land-surface initialisation improves the skill, although with larger differences between the forecasts started in May and the ones in June (the latter generally exhibiting higher skill, Fig. S4a). This is related to the choice of the target season (June-July-August) for computing the *CSI* and the temporal proximity to the initial conditions^30^.

**Figure 3:**
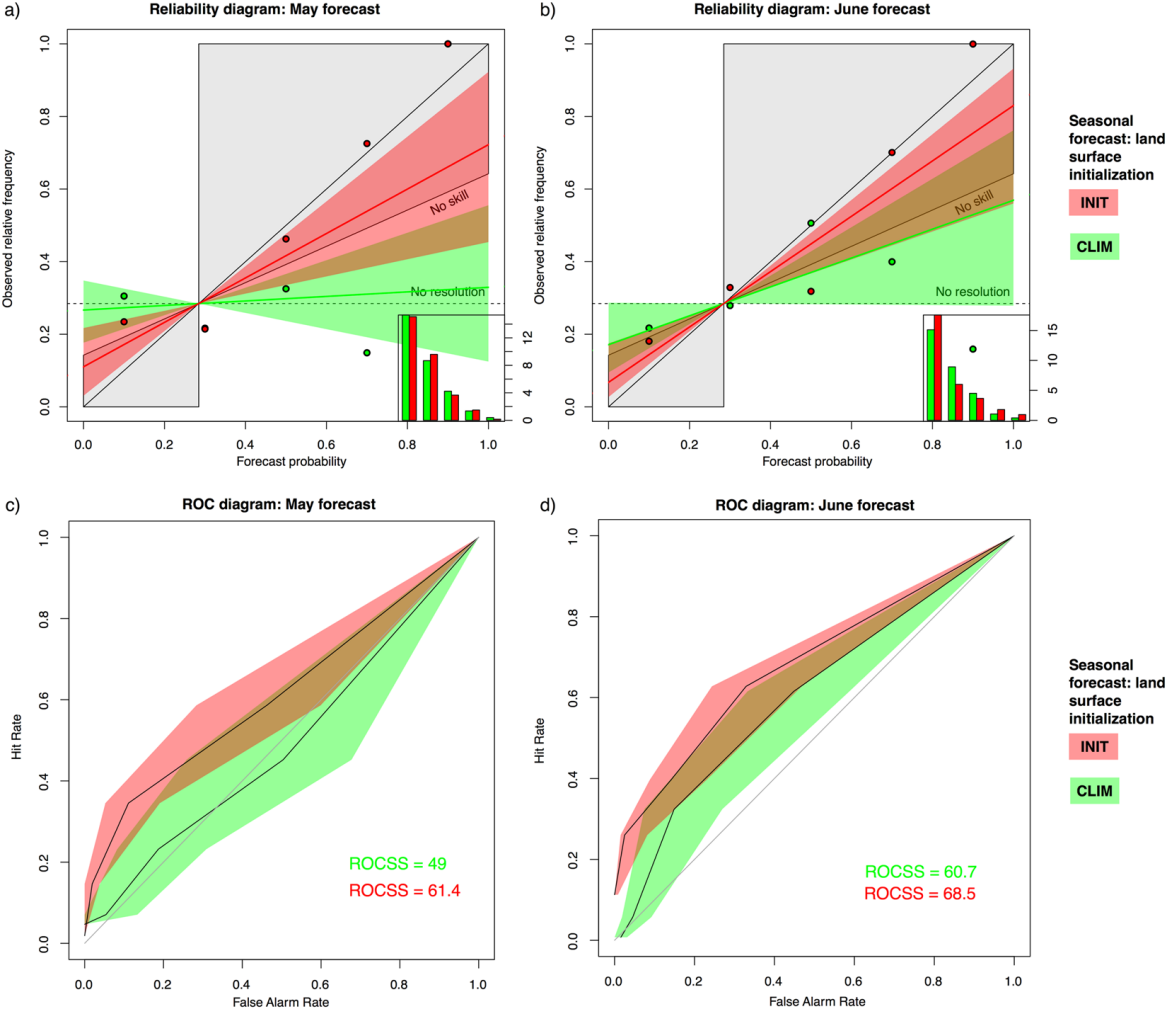
Skill of the seasonal climate forecasting system. (**a**) Reliability diagrams for low yielding events (*CSI*_*low*_, i.e. below the 25^*th*^ percentile) for May and June runs, driven by seasonal climate forecasts with climatological and realistic land-surface initialisations. The coloured lines show the linear weighted regression with the associated 75% confidence level (shaded areas). The number of samples for each bin is shown in the lower right sharpness diagram. The reliability diagram has been calculated by grouping the seasonal *CSI*_*low*_ forecasts for all countries having significant predictive performance under observed climate conditions. The horizontal and vertical lines indicate the climatological frequency of the events in the observations and forecasts, respectively. The grey area defines a region where seasonal *CSI*_*low*_ forecasts contribute positively to the forecast skill with respect to the climatology (the area where the Brier Skill Score is greater than 0^43^). The *n*o skill line separates skilful regions from unskilful ones in the diagram. The deviation from the diagonal provides the conditional bias; the flatter the curve, the less resolution it has (i.e. lower ability of the system to produce reliable forecasts that differ from the *n*aive probability). (**b**) ROC diagrams for low yielding events (i.e. below the 25th percentile) for May and June forecasts of *CSI*, driven by seasonal climate forecasts initiated by climatological and realistic land-surface initialisation. The shaded regions indicate the 75% confidence intervals calculated through 1000 bootstrap replications. The hit rate and false alarm rate values consider a set of probability forecasts by stepping a decision threshold with 20% probability through the forecasts. Each ROC diagram displays as well the ROCSS values for both *INIT* and *CLIM* forecast experiments.

We further examine the impact of the land-surface initialisation on the prediction of low yield events (*CSI*_*low*_), corresponding to the lower quartile of observed *CSI* (i.e. below the 25th percentile, computed from 30 years of *CSI* under observed climate conditions). For this purpose we use the reliability diagram, providing a visual assessment of probabilistic forecasts reliability (Fig. 3a,b). A perfectly reliable system should draw a line as close as possible to the main diagonal. Seasonal forecasts of *CSI*_*low*_ events driven by *INIT*05 and *INIT*06 exhibit reliability lines with associated uncertainty range within marginally useful limits for decision making^31^, as these forecasts carry a partial positive relationship between the model forecast probability and the observed frequency of occurrence of the event. On the other hand, *CLIM*05 shows no relationship between the forecast probabilities and the frequencies of the observed events. *CLIM*06 forecasts slightly improve over *CLIM*05, although with poorer skill than the *INIT*06 forecasts. The ROC diagram in Fig. 3c,d provides complementary information to the reliability diagram, since it is conditioned on observations (i.e. measures the ability of the forecasts to discriminate between two alternative outcomes). Clearly, *INIT*05 and *INIT*06 outperform *CLIM*05 and *CLIM*06, as also shown by the *ROCSS* values. It is worth noting that *CSI* seasonal forecasts driven by the climatological land-surface initialisation in June outperform a climatology-based forecast.

**Figure 4:**
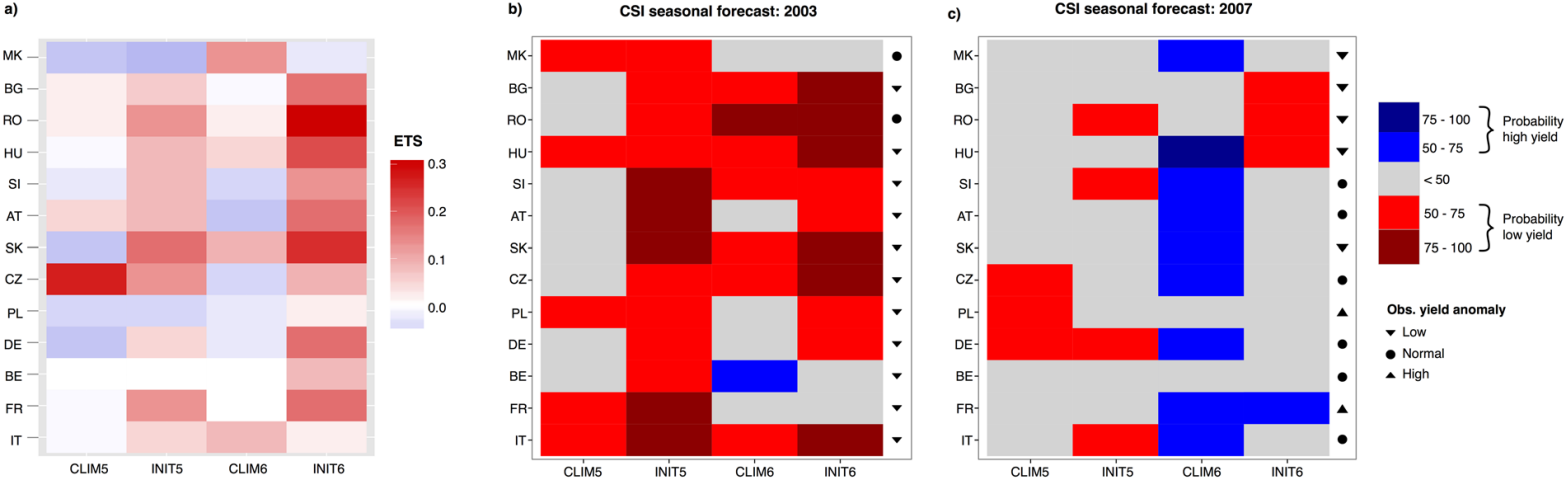
Seasonal forecasts for the 2003 and 2007 events. (**a**) Country-specific Equitable Threat Score (*ETS*) for each *CSI* forecast experiment. Low yielding event is defined each time at least 50% of initial condition ensemble members result in *CSI* belonging to the lower quartile range. Scores above 0 (equal to or below 0) indicate skill (no skill). (**b**) Forecast probabilities for low (*CSI* in the lower quartile range) and high (*CSI* in the upper quartile range) yielding events in 2003. The red and blue shading indicate the probability of *CSI* being very different from normal levels. The intensity of the colour indicates the probability of such an event occurring in summer, following the forecast initialisation. (**c**) Same as (**b**), but for 2007.

The skill estimates based on the past forecasting system performance may guide end-users on the expected performance of future forecasts. Therefore, we also compare the ability of the system to forecast two *CSI*_*low*_ events in 2003 and 2007. In order to provide a country-specific skill measure on how the forecasts correspond to the observed *CSI*_*low*_ events, the Equitable Threat Score (*ETS*) is calculated for *CSI*_*low*_ forecasts in each country. Figure 4a shows the derived *ETS* assuming that an event is correctly forecast each time at least 60% of ensemble members predict *CSI*_*low*_ (Fig. S5 provides similar graphs for other thresholds). In most of the cases the forecasts based on realistic land-surface initialisation outperform the ones based on climatological initialisation, confirming the aforementioned overall results. Additionally, *INIT*06 generally outperforms *INIT*05. However, it should be noted that also in the case of *INIT*06, the best *ETS* (identified in south-eastern Europe) still indicates rather moderate forecast skill, as the *CSI*_*low*_ events are correctly predicted in approximately one third of the cases (regardless of the choice of the probability threshold defining the event).

In 2003, the observed yield anomalies are in the lower quartile range in all countries but Macedonia and Romania (Fig. 4b). *INIT*05 predicts an anomalous event already in May in all countries with probability higher than 50% (70% in western Europe and several central European countries). The forecast probability of low yield event increases using the forecast initialised in June in south-eastern Europe, however the event is not anymore predicted in France and Belgium. While drought and heat wave are correctly forecast in Romania (not shown), their magnitude is overestimated, leading to the *CSI* forecast in the lower quartile range. The role of soil moisture initialisation in 2003 has been extensively studied^32^ and it is also confirmed by these findings.

In 2007, south-eastern Europe experienced severe summer drought and heat wave events^33^, resulting in substantially negative maize yield anomalies (Fig. 1a). *CLIM*06 fails to predict yields being in the lower quartile range in south-eastern Europe, while the opposite signal is given by *INIT*06 (Fig. 4c). Indeed at the time of forecast initialisation in May and June, the soil moisture levels were depleted due to the persisting drought from the preceding winter in most of central and south-eastern Europe. This example clearly demonstrates the importance of realistic land-surface initialisation for agricultural forecasting in south-eastern Europe. These findings are supported by previous assessments of realistic versus climatological soil moisture initialisations, indicating that forecast systems better simulate the warmest summers over south-eastern Europe when these events follow pronounced dry initial anomalies^18^. Considering the rest of Europe, *CLIM*06 generally fails to predict yield anomalies, except in France where high yield anomaly is forecast. *INIT*06 correctly captures the high yield anomaly in France, but not in Poland. Moreover, low yield anomalies in Slovakia and Macedonia are not accurately forecast.

## Conclusions

This study does not only provide a predictability assessment of both drought and heat stress events relevant for maize yields in Europe, but it also demonstrates how a proper land-surface initialisation in a seasonal climate forecast system can bring skill improvement in countries where a climatological land-surface initialisation fails. Given the still rather poor-to-moderate reliability of seasonal *CSI* forecasts, further efforts are clearly necessary to increase the skill of relevant agro-climatological predictors in Europe during summer. However, this study can serve as a baseline for future analyses including other experimental efforts to improve seasonal climate forecasts, such as increase in spatial resolution^16^. Additionally, other types of predictor variables, such as large-scale atmospheric patterns influencing crop yields^4^ and new skilful drought prediction methods, generated by combining dynamical seasonal forecasts with monitored data^34^, could be investigated to gain more seasonal predictability.

We would also like to emphasise that the maize sensitivity to heat and drought stress depends on factors such as agro-management practices and selection of varieties. Here, the *CSI* models are calibrated and validated on crop yield data between 1981 and 2010. By assuming stationarity in the identified relationship, such a model could be realistically used for, e.g., next year(s) forecasts. However, at longer time scales and under adoption of different adaptation strategies a new calibration would be required. More detailed spatial assessment is hindered by the use of national crop yield data; subnational data would be necessary to better capture the region-specific link between crop yield and climate variability and to perform a better seasonal forecast skill assessment.

## Methods

The *CSI*^19^ integrates the standardised precipitation evapotranspiration index SPEI^35^ and the heat magnitude day index *HMD*^19^. The *SPEI* is a multi-temporal-scale index quantifying persistent anomalies in the soil water balance over different time periods. The *SPEI* is able to capture the impact of drought on European agricultural production^36^. To consider the influence of heat stress and heat-related sub-optimal conditions for grain maize, we take into account a modified version of the *HMD* index, named heat degree days (*HDD*), based on the active temperature sum above a threshold temperature *T*_*thr*_, here 30 °*C*:

1 $$HD{D}_{JJA}=\sum _{i=1}^{N}\,{\max }\{{T}_{{\max }{,}i}-{T}_{thr},0\}$$

where *N* represents the number of summer days (June, July, August), coinciding with the sensitive stages of flowering and grain filling. As *T*_*thr*_ is chosen conservatively (i.e. close to the optimum temperature for growth processes^37^), the *HDD* incorporates the impact of a wide temperature range above the *T*_*thr*_ on growth processes in maize, which are deteriorating with increasing temperatures.

Our analysis is based on maize yield anomalies Y^*^ and anomalies of *SPEI* and *HDD* (hereafter SPEI^*^ and HDD^*^), obtained by de-trending their long-term time series. National crop yield time series have been obtained from national statistical institutes in Europe^26^. The study period spans between 1981 and 2010; only time series with at least 20 years of data are included in this study (Fig. 1a), as a tradeoff between having long enough time series of crop yields for statistical analysis and largest possible number of countries included in the analysis. A decadal trend in crop yield time series is usually an effect of changes in agro-management practices, environmental and socioeconomic factors and climate change. Therefore, polynomial method using linear and quadratic terms is applied on *log*(*yield*) to obtain the anomalies Y^*^^ 4^. Having potentially removed in this way also part of the climate signal, we are compelled to apply the same procedure also to the *HDD* and *SPEI* time-series. In such a way, we can isolate the effects of climate anomalies and extremes on the year-to-year maize yield variability. The Mann-Kendall test has been used to identify the presence of a trend.

Then, the *CSI* is defined as a simple linear combination of HDD^*^ and SPEI^*^:

2 $${Y}_{t}^{std{,}^{\ast }}=a\times SPE{I}_{opt,t}^{\ast }+b\times HD{D}_{JJA,t}^{std{,}^{\ast }}+{\varepsilon }_{t}=CS{I}_{t}+{\varepsilon }_{t}$$

where *t* indicates the year and the superscripts indicate whether variable is de-trended (*) and/or standardised (*std*). *Y* and *HDD* are de-trended and standardised, while SPEI is de-trended only as it is standardised by definition. Thus, the *CSI* is an estimate of the standardised maize yield anomalies. The regression coefficients *a* and *b* are obtained by maximising the predictive performance with a bilinear ridge regression on the observed yield anomalies Y^*^ at the national level, thus accounting for the covariance of the two explanatory agro-climatic indicators. The resulting multiplicative coefficients combining $HD{D}_{JJA}^{std{,}^{\ast }}$ and $SPE{I}_{opt}^{\ast }$ into the *CSI* are country dependent (Fig. S3). The subscript *opt* indicates the additional level of model optimisation introduced at country level. Different time scales for computing the *SPEI* have been tested in equation 2, from one to three months (i.e. *SPEI*1, *SPEI*2 and *SPEI*3), for each of the summer months. The optimal temporal aggregation period is identified by maximising the explained variability between leave-one-out *CSI* predictions and observed yield anomalies^4^, thus maximising the predictive performance of *CSI* under observed climate conditions. In this way, the period (during summer) having maximum sensitivity to drought stress is identified. Clearly, this is country specific due to spatial differences in varieties, agro-management practices (e.g. irrigation) and other socioeconomic factors. The results of this optimisation are shown in Fig. S1.

$SPE{I}_{opt,t}^{\ast }$ and $HD{D}_{JJA,t}^{std{,}^{\ast }}$ are derived at country level by using a grain maize crop mask^38^, i.e. estimating the spatial average weighted by the area of harvested maize in each country. The *CSI* models are derived from observational meteorological data, needed for the calculation of both predictands. For this purpose, the gridded meteorological dataset *E*−*OBS* (version 14.0)^39^ is used.

To validate the empirical estimates of maize yields based on *CSI*, the predictive performance (as measured by the *Q*^2^) is calculated by performing a leave-one-out cross validation on the available country-specific crop yield time series:

3 $${Q}^{2}=1-\,\frac{{\sum }_{t=1}^{M}{({\hat{y}}_{t}^{(-t)}-{y}_{t})}^{2}}{{\sum }_{t=1}^{M}{({y}_{t}-{y}_{mean})}^{2}},$$

where *M* represents the number of years, ${\hat{y}}_{t}^{(-t)}$ the yield predicted for year *t* without using *y*_*t*_ (using Eq. 2 calibrated on the remaining *M* − 1 years), *y*_*t*_ the observed yield anomaly in year *t* and *y*_*mean*_ the long-term average.

The seasonal re-forecast experiment is conducted with the EC-Earth2.3^29^. To assess the impact of a realistic land-surface initialisation on sub-seasonal and seasonal forecasts, two re-forecast experiments are performed: 10-member initial condition ensemble of 4-month long forecast experiments over the period 1981–2010 starting each year the first of May and the first of June^16^. In the *INIT* experiment, the land-surface is initialised with soil moisture, temperature and snow data from ERA-Interim Land^40^. The initial condition ensemble is constructed by using atmospheric singular vectors and the five ocean analyses available from ORAS4. The *CLIM* experiment initialises the land-surface using the climatology of ERA-Interim Land for the corresponding starting date, this being the only difference between *INIT* and *CLIM*. With this set up, the impact of the land-surface initialisation can be isolated from all the other factors influencing the quality of seasonal climate forecast. Four different seasonal *CSI* re-forecasts are obtained from *INIT* and *CLIM* with starting dates in May (*INIT*05 and *CLIM*05) and June (*INIT*06 and *CLIM*06).

A bias correction based on non-parametric quantile mapping is then applied^41^. Three meteorological variables are bias corrected to derive the *CSI*: monthly precipitation, average monthly temperature and maximum daily temperature in the period June-July-August.

Besides correlation coefficients we derive reliability diagrams, a common diagnostic tool for probabilistic forecasts showing for a specific event the correspondence of the predicted probabilities with the observed frequency of occurrence^31^. The events are here defined by the *CSI* dropping below the 25th percentile, calculated from the 30-year time series (*CSI*_*low*_). We also consider the ROC skill score (*ROCSS*), which is based on the area under the curve in the relative operating characteristics diagram (ROC). This diagram shows the hit rate (i.e. the relative number of times a forecast event actually occurred) against the false alarm rate (i.e. the relative number of times an event had been forecast but did not actually happen) for different potential decision thresholds. In order to have a large sample of probability forecasts, the reliability and ROC diagrams are computed by aggregating the country based forecasts, following the procedure recommended by the WMO^42^. Then, the probability forecasts are grouped into 5 bins and the observed occurrences/non-occurrences of *CSI*_*low*_ events are counted. Finally, the sum of counts is calculated using country specific maize cropland area weighting. The uncertainty of reliability slope and *ROCSS* is estimated by a bootstrap algorithm with replacement, randomly drawing from the set of forecast and observation data pairs, repeating the procedure 1000 times. 75% confidence interval of the resampling distribution is then used to define an uncertainty range around best-guess reliability slope and *ROCSS*.

The Equitable Threat Score (*ETS*^43^) is used as country-specific skill measure on how the forecasts correspond to the observed *CSI*_*low*_ events. The *ETS* provides a way of summarising the ability of a deterministic prediction to forecast a dichotomous event correctly. The score 1 is assigned to a perfect forecast, while random forecasts get a value equal to 0. The *ETS* measures the fraction of observed and/or forecast events that are correctly predicted, adjusted for hits associated with random chance:

4 $$ETS=\frac{j-{j}_{r}}{j+k+l-{j}_{r}},$$

where *j* represents the number of hits (events forecast to occur that did occur), *k* the number of false alarms (events forecast to occur that did not occur), *l* the number of misses (events forecast not to occur that did occur). The *j*_*r*_ is the expected fraction of hits for a random forecast:

5 $${j}_{r}=\frac{(j+l)(j+k)}{j+k+l+m}.$$

where *m* is the number of correct negatives (events forecast not to occur that did not occur). Here, *ETS* is calculated for different probability thresholds, assuming that a *CSI*_*low*_ event is forecast each time at least 50% (60%, 70%, 80%) of the ensemble members predict *CSI*_*low*_ (Fig. S5).

R-software has been used for data analysis and creating all graphs^44^.

## Electronic supplementary material

Supplementary material
