# Supplementary material for: Land-surface initialisation improves seasonal climate prediction skill for maize yield forecast
Source: Sci Rep. 2018 Jan 22;8:1322. doi: 10.1038/s41598-018-19586-6 (PMC5778075; doi:10.1038/s41598-018-19586-6)
Supplement: Supplementary file 1 — Supplementary material [file 41598_2018_19586_MOESM1_ESM.pdf]

Supplementary material for: Land-surface initialisation improves seasonal climate prediction skill for maize yield forecast

Andrej Ceglar[1,*], Andrea Toreti[1], Chloe Prodhomme[2], Matteo Zampieri[1], Marco Turco[3], and Francisco J. Doblas-Reyes[2,4]

[1]European Commission, Joint Research Centre, via Enrico Fermi 2749, 21027, Ispra, Italy
[2]Barcelona Supercomputing Center (BSC), c Jordi Girona 29, 08034, Barcelona, Spain
[3]University of Barcelona, Av. Diagonal 647, 08028, Barcelona, Spain
[4]Institució Catalana de Recerca i Estudis Avançats (ICREA), Passeig de Lluis Companys 23, 08010, Barcelona, Spain
[*]andrej.ceglar@ec.europa.eu

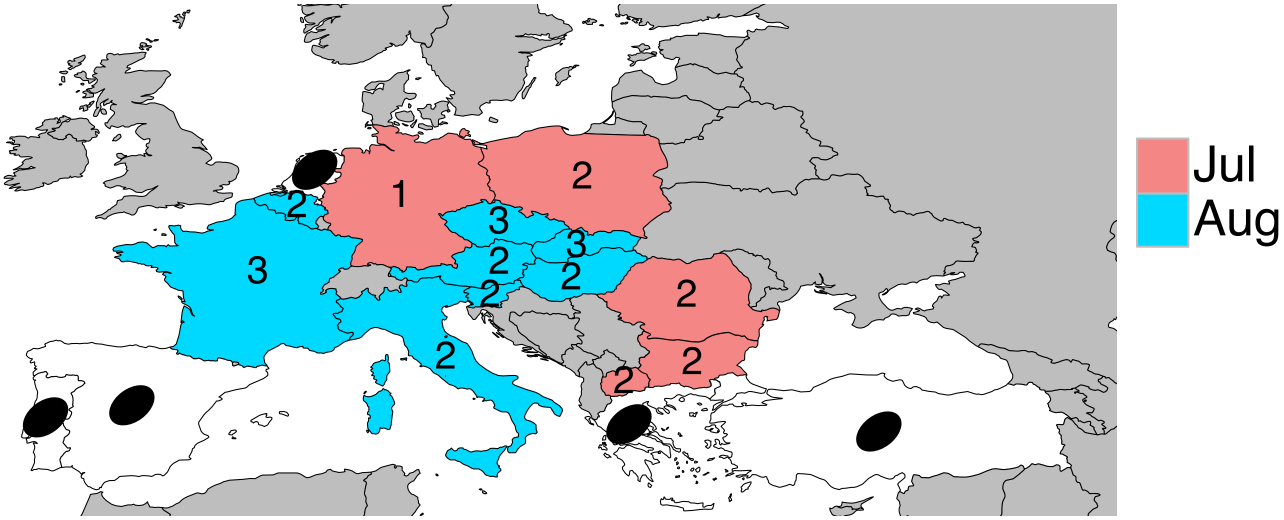

Figure S1. An optimal month (represented by color) and time scale (represented by number for each country) of $SPEI_{opt}$ predictor, entering equation 2 in the main paper, as selected by optimization procedure, which maximizes the predictive performance and minimizes the mean square error on Leave-One-Out predictions of standardized maize yield anomalies ($Y^{*,std}$). As an example, *SPEI* for August, aggregating the precipitation and temperature conditions during the 3 preceding months (June-July-August) has been selected for France. Black ellipses indicate countries, where the *CSI* model is not significant. R software has been used to produce the figure (R Core Team, 2017). R package *ggmap* (Kahle and Wickham, 2013) has been used for spatial visualization and administrative boundaries.

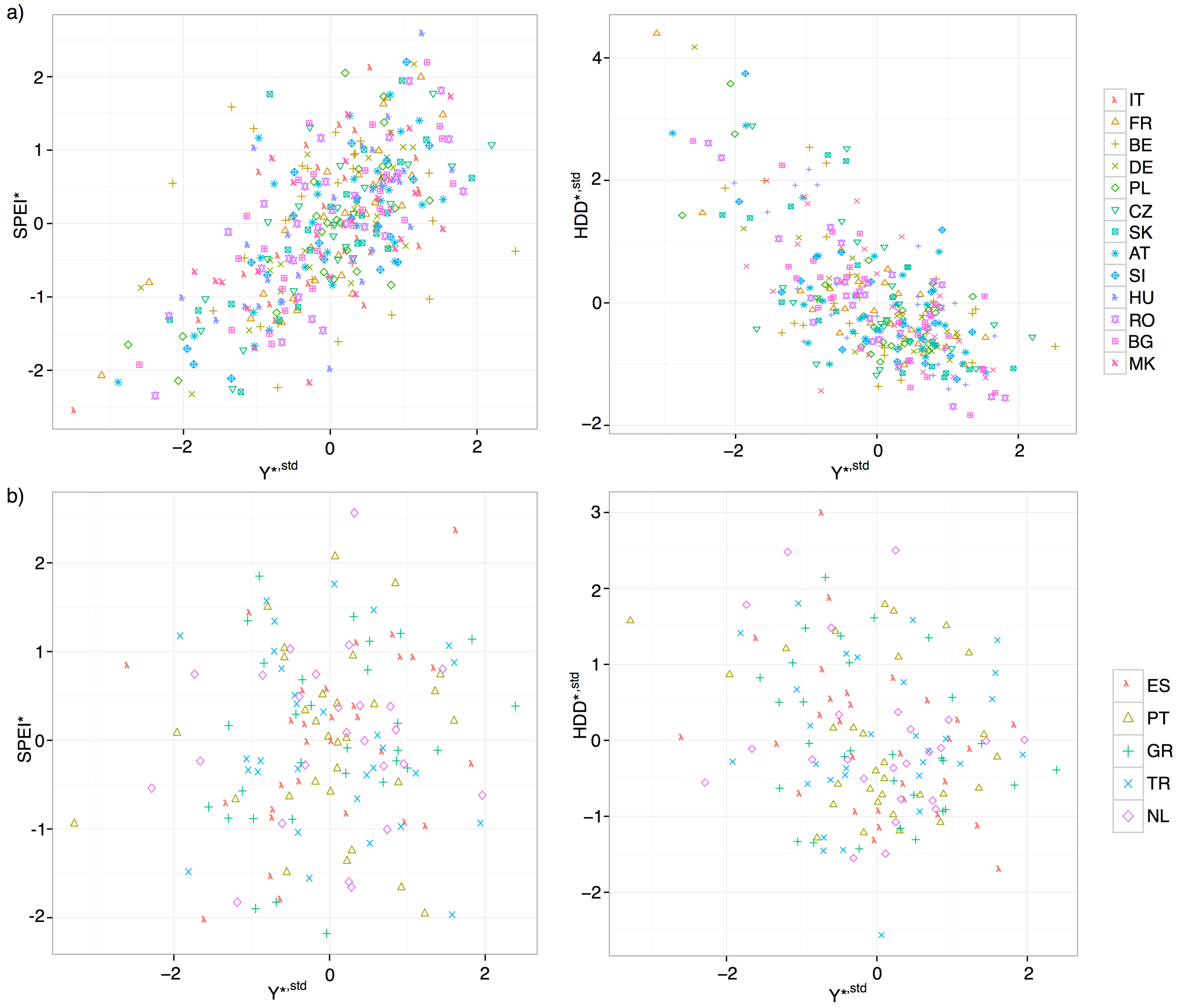

Figure S2. a) Scatterplots between predictand ($Y^{*,std}$) and both predictors (detrended and standardized Heat Degree Days - $HDD^{*,std}_{JJA,t}$, and detrended standardized precipitation evapotranspiration index - $SPEI^{*}$), entering the regression equation 2 of the main paper, in analysed countries where a substantial proportion of maize is rainfed (i.e. more than 60 % according to the state in 2010; Eurostat, 2017). b) Same as a), but for analysed countries where irrigated maize prevails over rainfed: Spain, Portugal, Greece and Turkey (Portmann et al., 2010), and the Netherlands, where derived *CSI* statistical model is not significant.

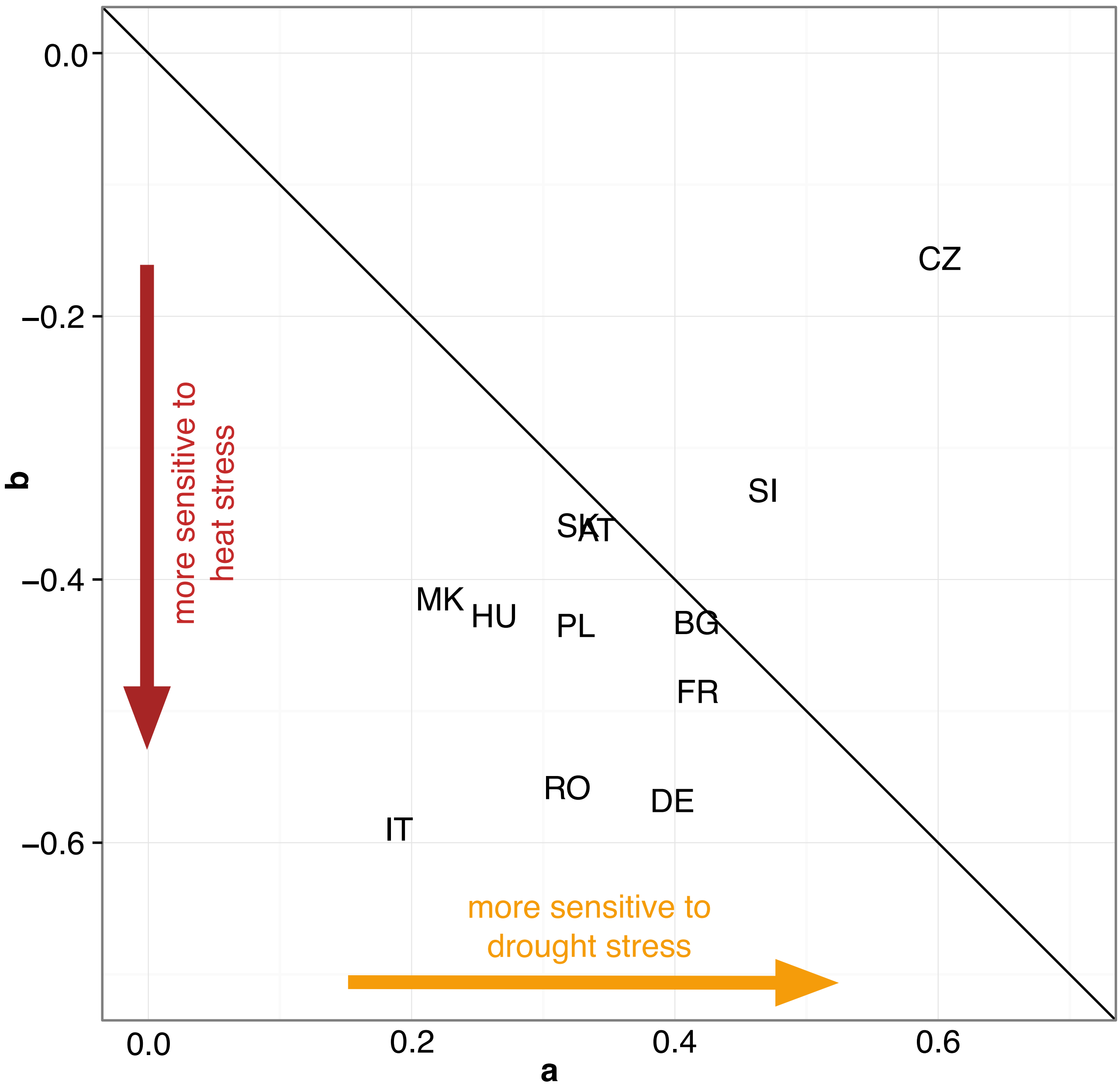

Figure S3. Scatterplot between country specific standardized regression coefficients *a* and *b* (see equation 2 of the main paper). Regression coefficient *a* provides an estimate of the effects of drought stress, whereas coefficient *b* an estimate of heat stress, in determining the maize yield anomalies, respectively. The sign of *a* (*b*) is always resulting negative (positive), indicating that increasing heat and drought stress negatively affect crop yields. The sensitivity of yield anomalies to heat and drought stress differ in analysed countries; the arrows indicate the direction of increasing sensitivity to heat stress (red) and drought stress (orange). The black line indicates the values of equal sensitivity to heat and drought stress. Due to standardization, the regression coefficients are comparable between different countries.

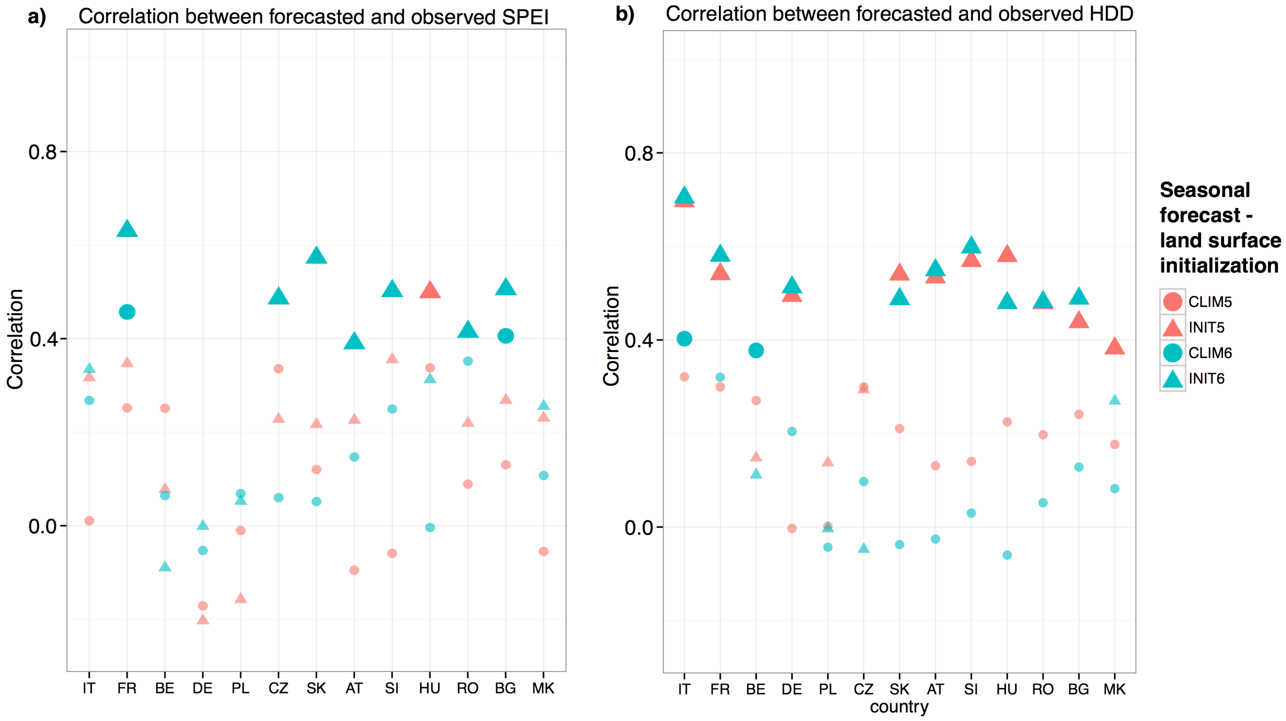

Figure S4. a) Pearson correlation between country specific $SPEI^{*}_{opt}$ (see Supplementary Fig. 1) calculated from EOBS observational data and different seasonal forecast experiments using climatological ($CLIM05$ and $CLIM06$ for May and June initialisations, respectively) or realistic land surface initialisation ($INIT05$ and $INIT06$ for May and June initialisations, respectively). The forecasted $SPEI^{*}_{opt}$ has been calculated from initial condition ensemble average for each of the forecast experiments. The significance of correlation is indicated by the size of symbols; larger size indicates significant correlations ($p<0.05$), whereas smaller size indicates non-significant correlations. b) Same as left, but correlations are calculated between forecasted and observed $HDD^{*,std}_{JJA}$.

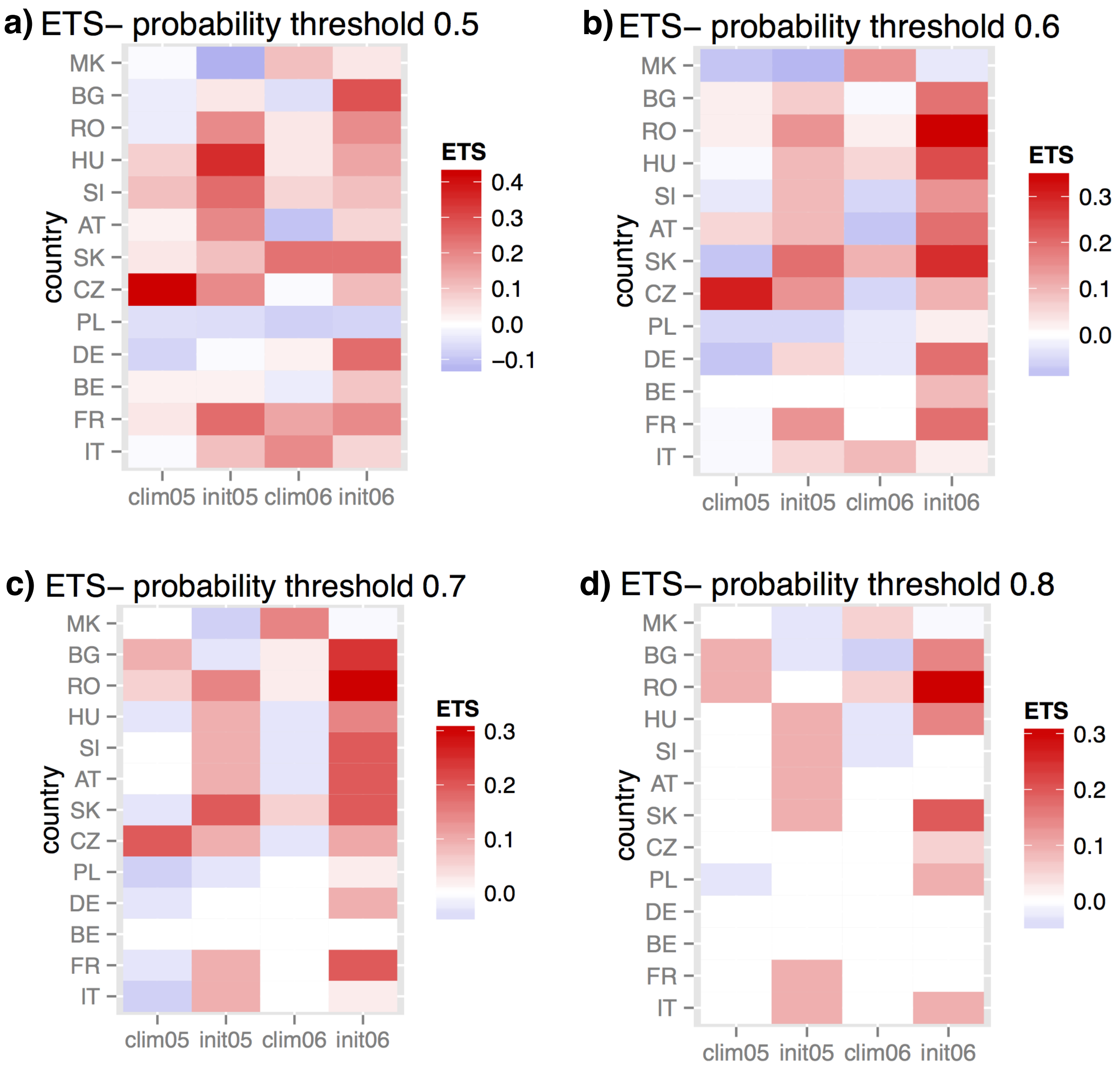

Figure S5. Country-specific Equitable Threat Score (ETS) for each *CSI* forecast experiment. Low yielding event is defined each time at least a) 50%, b) 60 %, c) 70% and d) 80% of initial condition ensemble members result in *CSI* belonging to the lower quartile range (i.e. below 25th percentile of observed *CSI* time series). Scores above 0 (equal to or below 0) indicate skill (no skill).

References

1. Eurostat - Agriculture, forestry and fisheries database (European Commission database). http://ec.europa.eu/eurostat/data/database (2017).
2. Kahle, D. & Wickham, H. ggmap: Spatial Visualization with ggplot2. *The R Journal* **5(1)**, 144-161 (2013).
3. Portmann, F.T., Siebert S. & Döll, P. MIRCA2000-Global monthly irrigated and rainfed crop areas around the yer 2000: A new high-resolution data set for agricultural and hydrological modelling. *Global Biogeochem. Cy.* **24**, GB1011 (2010).
4. R Core Team. R: A Language and Environment for Statistical Computing. R foundation for statistical computing, https://www.R-project.org (2016).
